# Echocardiographic Parameters in the Assessment of Tricuspid Regurgitation Prognosis: What Is New?

**DOI:** 10.3390/jcm15218588

**Published:** 2026-11-04

**Authors:** Bianca Rocca, Javier Solsona-Caravaca, Martin Roncaglia, Nuria Vallejo, Víctor González Fernández, Pablo E. Tobías-Castillo, Gisela Teixidó-Tura, Ruper Oliveró-Soldevila, Guillem Casas, Filipa Valente, Oro Aran, Ignacio Ferreira-González, José Rodríguez-Palomares, Laura Galian-Gay

**Affiliations:** 1 Department of Cardiology, Vall d’Hebron Hospital Universitari, Vall d’Hebron Barcelona Hospital Campus, 08035 Barcelona, Spain; biancarocca94@gmail.com (B.R.); martinroncaglia01@gmail.com (M.R.); nuria.vallejo@vallhebron.cat (N.V.); victor.gonzalez@vallhebron.cat (V.G.F.); gisela.teixido@vallhebron.cat (G.T.-T.); rupertocarlos.olivero@vallhebron.cat (R.O.-S.); guillem.casas@vallhebron.cat (G.C.); filipaxaviervalente@gmail.com (F.V.); oroaran1606@gmail.com (O.A.); ignacio.ferreira@vallhebron.cat (I.F.-G.); laura.galian@vallhebron.cat (L.G.-G.); 2 Cardiovascular Diseases, Vall d′Hebron Institut de Recerca (VHIR), Vall d’Hebron Hospital Universitari, Vall d’Hebron Barcelona Hospital Campus, Passeig Vall d’Hebron 119-129, 08035 Barcelona, Spain

**Keywords:** tricuspid regurgitation, echocardiography, prognosis, right ventricular function, right ventricular–pulmonary artery coupling, transcatheter tricuspid valve intervention

## Abstract

Tricuspid regurgitation (TR) is associated with adverse clinical outcomes, highlighting the need for echocardiographic assessment beyond conventional severity grading. This narrative review examines the prognostic value and clinical applicability of established and emerging echocardiographic parameters. TR phenotype influences prognosis and response to transcatheter intervention, with more favourable outcomes in atrial than ventricular functional TR. The five-grade TR classification improves risk stratification, with massive or torrential TR associated with worse outcomes despite comparable procedural success. Right ventricular–pulmonary arterial coupling provides valuable prognostic information and may help identify an optimal therapeutic window for transcatheter intervention, particularly in patients with intermediate coupling values. The GLIDE score helps predict anatomical suitability and procedural success for transcatheter edge-to-edge repair, whereas high TRI-SCORE values (≥6) identify patients in whom a significant survival benefit from repair has not been demonstrated. Treatment decisions should therefore integrate symptoms, overall clinical status, procedural feasibility, and expected benefit through individualized Heart Team assessment. Following intervention, the severity of residual TR, ideally mild or less, remains a key determinant of procedural success.

## 1. Introduction

The tricuspid valve has emerged as a major focus of contemporary valvular heart disease research. Once considered the “forgotten valve”, tricuspid regurgitation (TR) is now recognized as a highly prevalent condition associated with major prognostic implications. Moderate or greater TR is observed in up to 25% of individuals over 75 years of age [1]. Its burden is particularly pronounced among older individuals with left-sided heart disease, and it is more frequent in women than in men [2]. Moderate-to-severe TR is associated with adverse clinical outcome, including increased hospitalization from heart failure and reduced long-term survival [3,4,5]. Accordingly, accurate phenotyping and severity assessment have become increasingly important for prognostic stratification and therapeutic decision-making.

The advent of transcatheter tricuspid interventions has further expanded the role of cardiac imaging. Although cardiac computed tomography (CT) and cardiac magnetic resonance (CMR) have become increasingly important for procedural planning and right ventricular assessment, echocardiography remains the cornerstone of TR evaluation because of its ability to provide comprehensive assessment of valve anatomy, disease mechanism, right-heart remodelling, and regurgitation severity. Beyond diagnosis, echocardiography has become central to risk stratification, treatment selection, procedural guidance, and longitudinal follow-up.

**Figure 1:**
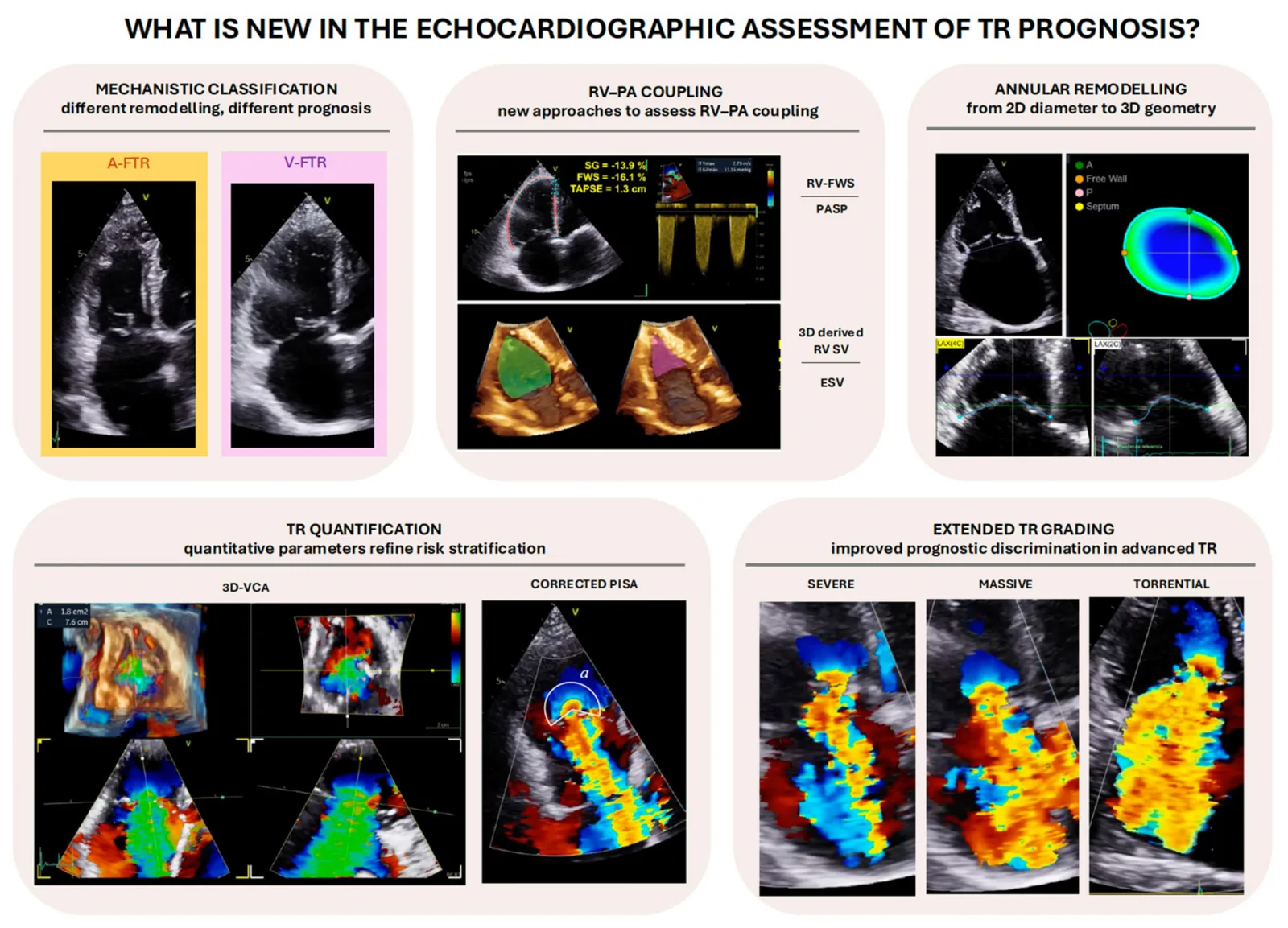
Novel echocardiographic parameters in tricuspid regurgitation assessment.

The present review focuses on the prognostic significance of emerging echocardiographic parameters, with particular attention given to three-dimensional (3D) imaging. Their incremental clinical and prognostic value beyond conventional parameters, together with their methodological limitations, is critically examined. In addition, a patient-centred perspective is considered, with the aim of exploring how echocardiographic findings may contribute to treatment selection and timing, an important unresolved challenge in the management of TR (Figure 1).

A targeted literature search was conducted in PubMed/MEDLINE, covering publications from January 2005 to September 2026. Representative search terms included “tricuspid regurgitation”, “tricuspid valve insufficiency”, “echocardiography”, “three-dimensional echocardiography”, “vena contracta area”, “proximal isovelocity surface area”, “right ventricular function”, “right atrial strain”, “right ventricular–pulmonary arterial coupling”, “TAPSE/PASP”, “prognosis”, “mortality”, “heart failure hospitalization”, and “transcatheter tricuspid intervention”. Relevant original studies, observational registries, randomized clinical trials, systematic reviews, clinical practice guidelines, and consensus documents were considered based on their relevance to the scope of this narrative review.

## 2. Mechanistic Phenotypes of Tricuspid Regurgitation

Distinct TR aetiologies are associated with different patterns of right-heart remodelling, disease progression, response to intervention, and clinical outcomes. This paradigm shift has generated the hypothesis that mechanistic classification may improve prognostic stratification beyond conventional severity grading [4,6,7].

Primary, or organic, TR represents the minority, accounting for about 5–10% of cases [8,9]. It is characterized by structural leaflet abnormalities of degenerative, congenital, or acquired origin [9,10]. Secondary, or functional, TR is the predominant form and results from alterations in the geometry of the tricuspid valve apparatus, leading to inadequate leaflet coaptation despite structurally normal leaflets. It may arise as a consequence of several conditions, including left ventricular dysfunction, left-sided heart disease, atrial fibrillation, and pulmonary hypertension [11]. More recently, a third category related to cardiac implantable electronic device (CIED) leads (lead-related TR) has been recognized [8]. CIED-associated TR has also been associated with an increased risk of the composite outcome of all-cause mortality and heart failure (HF) hospitalizations (adjusted hazard ratio [HR] 1.96, 95% confidence interval [CI] 1.33 to 2.87) [12].

Contemporary understanding of functional TR recognizes two major phenotypes: atrial functional TR (A-FTR) and ventricular functional TR (V-FTR). This distinction extends beyond pathophysiology, as accumulating evidence indicates that A-FTR and V-FTR represent clinically distinct entities, characterized by different patterns of right-heart remodelling, disease progression, and prognosis. Regardless of aetiology, TR leads to maladaptive right ventricular (RV) dilation, which, in turn, may cause further annular dilation and leaflet tethering. This self-perpetuating remodelling process progressively blurs the distinction between these phenotypes over time [9].

V-FTR is characterized by RV dilation and remodelling, predominantly involving the mid-portion of the RV free wall, which results in a more elliptical or spherical chamber geometry [5,9]. This remodelling disrupts the spatial relationship between the leaflets, chordae, and papillary muscles, leading to apical displacement of the papillary muscles, leaflet tethering [13], and subsequent malcoaptation [5,9]. Although tricuspid annular and right atrial (RA) dilation are also present, they are typically less pronounced than in the atrial form [14]. Progressive TR promotes further RV dilation, and systolic function gradually declines. Rising RV diastolic pressure and septal shift further perpetuate valvular distortion [15]. In most cases, RV enlargement and dysfunction are secondary to elevated pulmonary pressures, which may be post-capillary—due to left ventricular dysfunction or left-sided valvular disease—or pre-capillary in origin. A pulmonary artery systolic pressure ≥ 50 mmHg represents an additional parameter useful in the echocardiographic characterization of V-FTR [7]. However, a smaller subset of V-FTR cases occurs in the absence of pulmonary hypertension, typically in the context of primary RV cardiomyopathies [5,16]. Severe leaflet tethering is a strong predictor of recurrent TR after surgical repair. On two-dimensional (2D) echocardiography, a tethering height > 0.76 cm, or a tenting area > 1.63 cm^2^ has been associated with TR recurrence [17], while a 3D-derived tenting volume > 2.3 mL predicted residual TR after tricuspid annuloplasty with 100% sensitivity and 84% specificity [18,19,20].

A-FTR is most commonly associated with longstanding atrial fibrillation, although RA myopathy in patients with heart failure and preserved ejection fraction may also contribute [14]. A hallmark of A-FTR is the disproportionate degree of RA enlargement relative to RV remodelling. A-FTR is characterized by an increased end-systolic ratio between atrial and ventricular volumes (RA/RV volume ratio ≥ 1.4) as well as between their areas (end-systolic RA/RV area ratio ≥ 1.6) [16,21]. In A-FTR, in addition to RA and annular dilation, the right ventricle is typically normal in size or only mildly enlarged at the basal level, with preservation of its characteristic triangular/conical shape and overall function. As a result, leaflet tethering is usually mild or absent, and pulmonary hypertension is generally not present [9].

Compared with V-FTR, A-FTR is consistently associated with a more favourable prognosis and better response to transcatheter intervention. At 10 years, patients with A-FTR demonstrate significantly better survival compared with V-FTR (78% vs. 46%; *p* < 0.001) [7]. Similarly, in the TriValve registry, 12-month survival was 91% in A-FTR compared with 72% in V-FTR, while the EuroTR registry reported a higher 2-year survival free from heart failure hospitalization in A-FTR than in V-FTR (66.3% vs. 47.5%; *p* < 0.001) [22,23]. This survival advantage is likely explained by the relative preservation of RV geometry and systolic function in A-FTR, as RV dysfunction has consistently emerged as one of the strongest predictors of adverse outcomes in TR, which may limit the prognostic benefit of valve correction despite successful reduction in TR [22,24,25].

## 3. Tricuspid Annular Remodelling

Tricuspid annular remodelling plays a central role in the progression of TR and has important implications for both prognostic assessment and transcatheter procedural planning. Traditionally, TA size is assessed by 2D echocardiography using the end-diastolic diameter in the apical four-chamber view, with values ≥ 40 mm or ≥21 mm/m^2^ considered indicative of annular dilatation [10]. However, 2D assessment has important limitations. It primarily evaluates the septal-lateral diameter, whereas annular dilatation predominantly occurs along the anteroposterior axis. Furthermore, because of the lack of fixed anatomical landmarks, measurements are highly dependent on probe orientation and fail to capture the complex three-dimensional, non-planar, and dynamic geometry of the annulus throughout the cardiac cycle [13,26]. 3D echocardiography overcomes these limitations by enabling comprehensive quantification of annular area, perimeter, major and minor diameters, annular shape, and dynamic changes throughout the cardiac cycle [26,27]. Accordingly, TA dimensions should be reported in both systole and diastole, particularly when evaluating patients for transcatheter tricuspid valve replacement (TTVR), in whom accurate characterization of annular dynamics is relevant for device sizing and procedural planning.

Beyond anatomical characterization, TA remodelling also carries clinical and prognostic implication. Among patients undergoing left-sided valvular intervention, larger baseline TA diameter was linearly associated with subsequent TR progression and independently predicted mortality and heart failure hospitalization [28]. Furthermore, the TRILUMINATE imaging substudy demonstrated early and sustained annular reverse remodelling after successful transcatheter edge-to-edge repair (TEER), with an approximately 11% reduction in annular area at 30 days that persisted at 1 year, as assessed by serial CMR and four-dimensional CT. The magnitude of annular reverse remodelling was associated with the degree of TR reduction, supporting TA geometry not only as a marker of disease severity but also as a potential imaging marker of treatment response [29].

## 4. Contemporary Grading of TR Severity

**Figure 2:**
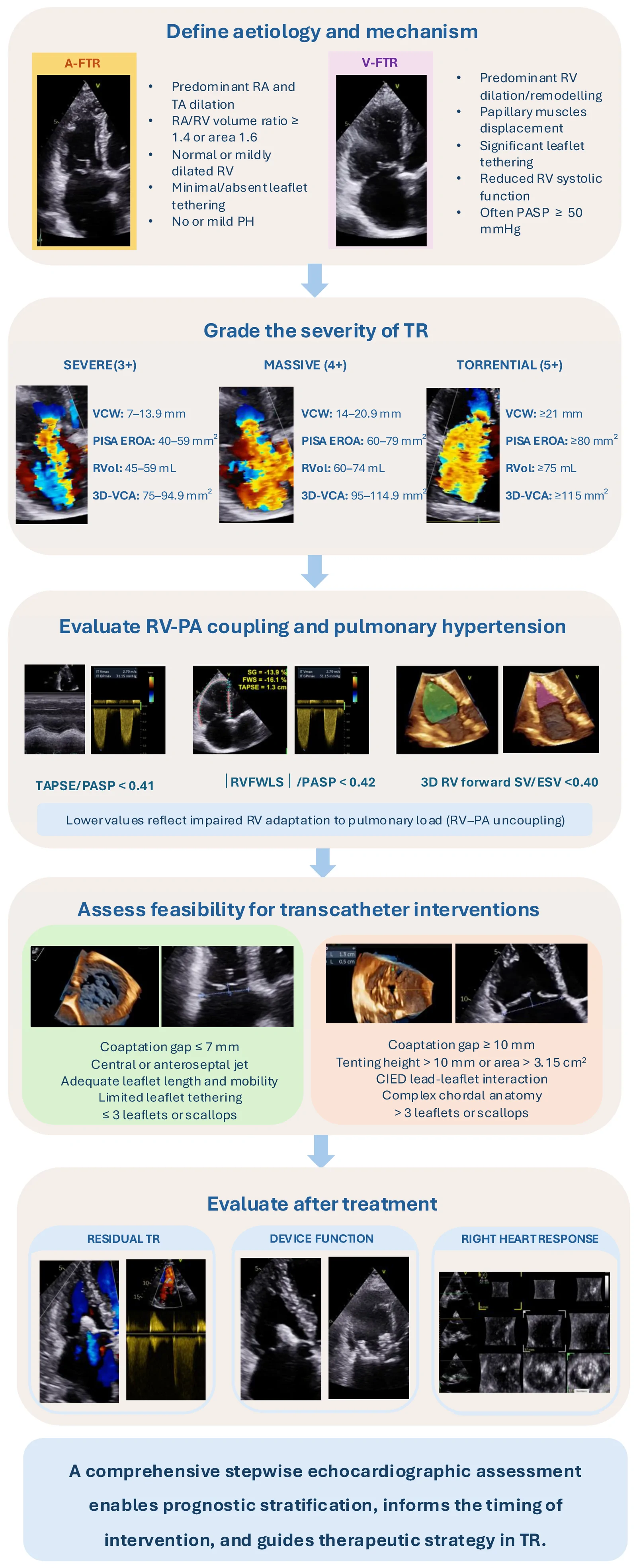
Stepwise echocardiographic assessment of tricuspid regurgitation.

A major advance in TR assessment is the extension of the conventional three-grade classification (mild, moderate, and severe) to a five-grade scheme. Severe TR has traditionally been defined by a vena contracta width (VCW) ≥ 0.7 cm, an effective regurgitant orifice area (EROA) ≥ 0.40 cm^2^, and/or a regurgitant volume ≥ 45 mL/beat. However, the absence of an upper boundary for the severe category became increasingly inadequate to characterize the full spectrum of advanced TR with the advent of TEER, as patients enrolled in early studies frequently exhibited regurgitation far exceeding these thresholds. The limitations of the conventional classification were further illustrated by the SCOUT trial, in which EROA decreased from 0.51 ± 0.18 to 0.32 ± 0.18 cm^2^ following intervention, accompanied by an increase in forward stroke volume and improvement in quality of life [30]. Thus, these findings highlighted the need for a more granular grading system capable of capturing the magnitude of TR reduction following intervention (Figure 2). To address this limitation, Hahn and Zamorano proposed in 2017 the addition of massive (4+) and torrential (5+) categories, extending quantitative grading beyond severe TR, with EROA ranges subsequently defined as 0.40–0.59, 0.60–0.79, and ≥0.80 cm^2^ for severe, massive, and torrential TR, respectively [31]. The five-grade scheme was subsequently endorsed by the Tricuspid Valve Academic Research Consortium (TVARC) for the assessment of TR reduction endpoints in transcatheter trials [9]. The application of this extended classification has subsequently shown that patients encompassed within the conventional severe category differ substantially in disease burden and prognosis. In TRILUMINATE, >70% of patients undergoing TEER had massive or torrential TR at baseline, illustrating the advanced spectrum of disease encountered in contemporary TEER populations [32,33]. The prognostic relevance of this classification was further supported by data from the TriValve registry. Despite similar procedural success rates compared with patients with severe TR (83.2% vs. 77.3%; *p* = 0.21), patients with massive or torrential TR had a nearly two-fold higher adjusted risk of all-cause death or heart-failure rehospitalization at 1 year (adjusted HR 1.91, 95% CI 1.10–3.34; *p* = 0.022). Importantly, among patients presenting with massive or torrential TR, procedural success (defined as successful device implantation and delivery-system retrieval with residual TR ≤ 2+) was associated with greater freedom from death or heart-failure rehospitalization (69.9% vs. 54.2%; *p* = 0.048) [32].

### 4.1. Integrated Echocardiographic Assessment of TR Severity

Classification of TR across the five-grade severity spectrum requires an integrated multiparametric echocardiographic approach, as no single measurement adequately reflects the complexity of the regurgitant lesion. Assessment should begin with qualitative features and be complemented by semiquantitative and quantitative parameters to achieve a comprehensive evaluation [10]. It should be emphasized that TR severity is highly sensitive to haemodynamic conditions. Volume overload may lead to an overestimation of TR severity. In addition, TR is influenced by the respiratory cycle, typically worsening during inspiration, which may introduce variability in measurements. Therefore, it is recommended to assess TR under near-euvolemic conditions and to average measurements over 5–10 cardiac cycles during quiet respiration [11,21].

### 4.2. Qualitative Assessment

Qualitative assessment of TR integrates valve morphology with Doppler-derived features, including jet size and eccentricity, flow convergence, and systolic flow reversal in the hepatic veins, and remains useful for the initial identification and characterization of TR [14]. Hepatic vein systolic flow reversal is a specific marker of haemodynamically significant TR; however, it is influenced not only by regurgitant severity but also by RA compliance, the systolic pressure gradient between the RV and RA, hepatic and portal venous haemodynamic, and cardiac rhythm. These confounding factors may limit its ability to accurately reflect immediate haemodynamic changes following TEER when assessing residual TR severity [14].

### 4.3. Semi-Quantitative Assessment

VCW remains one of the most widely used indices of TR severity. VCW is commonly measured in the apical four-chamber view. However, the resulting septo-lateral dimension frequently represents the minor axis of an elliptical regurgitant orifice and may therefore underestimate its true dimensions [9,11,21]. Current guidelines define severe TR as a two-dimensional VCW ≥ 7 mm [34]. Notably, among patients with severe TR, a VCW > 9 mm was associated with adverse RV remodelling and increased mortality [35]. Other VCW-derived parameters have also shown prognostic value. Fortuni et al. proposed the ratio of VCW to TA diameter (VCW/TA), with values ≥ 0.24 independently associated with increased mortality and lower 5-year survival (HR 1.57; 95% CI 1.044–2.352; *p* = 0.030) [36]. Qualitative and semi-quantitative indices are particularly useful for identifying mild TR but provide limited discrimination across more advanced stages. Therefore, quantitative assessment remains essential for characterizing the full spectrum of TR severity [14].

### 4.4. Quantitative Assessment

EROA is one of the most extensively validated quantitative indices for risk stratification in TR, with values ≥ 40 mm^2^ independently associated with mortality and cardiac events [3]. In a cohort of 382 patients with heart failure with reduced ejection fraction (HFrEF), Bartko et al. reported a continuous association between increasing EROA and PISA-derived regurgitant volume and mortality. In unadjusted analyses, the hazard ratios were 1.009 (95% CI 1.004–1.013; *p* < 0.001) per 0.01 cm^2^ increase in EROA and 1.013 (95% CI 1.007–1.020; *p* < 0.001) and per 1-mL increase in regurgitant volume [37]. The authors also identified EROA ≥ 0.20 cm^2^ and regurgitant volume ≥ 20 mL as thresholds associated with increased mortality [37]. In another cohort of 296 patients with functional TR, a regurgitant volume > 30 mL identified the highest-risk group for death or heart-failure hospitalization over 47 months [38].

These findings suggest that adverse clinical outcomes may occur at EROA and regurgitant volume values below the conventional thresholds for severe TR. However, their implications for therapeutic decision-making, particularly regarding the optimal timing of intervention, remain uncertain. The available evidence is predominantly derived from observational studies involving selected and heterogeneous populations with different TR etiologies and varying degrees of left ventricular involvement. Accordingly, prognostic thresholds identified in patients with functional TR cannot necessarily be extrapolated to those with primary TR or to populations with different underlying cardiac conditions. Furthermore, although PISA-derived quantitative parameters are attractive from a prognostic perspective, their clinical applicability remains limited by important methodological constraints.

In TR, the regurgitant orifice is frequently non-circular and non-planar, reflecting the complex anatomy of the tricuspid valve and its variable number of leaflets and commissures. Consequently, the hemispheric flow-convergence assumption underlying conventional PISA may result in systematic underestimation of EROA [21]. The increasing availability of 3D echocardiography has provided an important reference against which PISA-derived measurements can be evaluated. Using 3D volumetric assessment as the comparator, Tomaselli et al. demonstrated that conventional PISA underestimated secondary TR severity in approximately 50% of patients. Similarly, PISA-derived EROA has been reported to underestimate the 3D vena contracta area (VCA) by approximately 40%, depending upon the regurgitant orifice shape [38,39]. To overcome some of these limitations, a corrected PISA approach has been proposed to account for the geometric and haemodynamic characteristics of secondary TR. Correction for leaflet tethering angle and the relatively low regurgitant jet velocity addresses deviations from the assumptions underlying conventional PISA. These adjustments reduce the underestimation of regurgitant severity and improve the agreement of PISA-derived EROA and regurgitant volume with 3D volumetric assessment [39]. Beyond improving TR quantification, corrected PISA-derived parameters provide improved risk stratification for mortality and heart-failure hospitalization [40].

Three-dimensional echocardiography has also enabled direct planimetry of the VCA, providing a quantitative assessment of the regurgitant orifice without relying on the hemispheric flow-convergence assumptions inherent to PISA. Contemporary consensus frameworks have incorporated 3D-VCA into TR assessment and extended severity grading, with a VCA ≥ 0.75 cm^2^ proposed as the threshold for severe TR [9].

Although below this threshold, patients with a VCA > 0.65 cm^2^ experienced a threefold increase in the risk of all-cause death or heart failure hospitalization [41]. Importantly, 3D-VCA remained independently associated with outcomes and provided incremental prognostic value beyond EROA and regurgitant volume [41]. The incremental prognostic value of 3D-VCA may partly be explained by its ability to better characterize the complex and frequently non-circular geometry of the tricuspid regurgitant orifice, particularly in functional TR, where annular dilatation and leaflet tethering may produce irregular orifice configurations. Nevertheless, 3D-VCA remains a single time-point measurement and may therefore incompletely capture the dynamic nature of TR throughout systole. Furthermore, 3D colour Doppler is limited by relatively low temporal resolution and susceptibility to stitching artefacts with multibeat acquisitions, particularly in patients with arrhythmias.

Thus, no single echocardiographic parameter can currently be considered a standalone gold standard for TR quantification. An additional challenge lies in the clinical interpretation of quantitative parameters and their prognostic thresholds. Although several indices have demonstrated prognostic value, universally validated cut-offs for identifying high-risk patients remain lacking. Reported thresholds may vary according to TR etiology, disease stage, loading conditions, and imaging methodology, limiting their comparability and generalizability across different populations. Moreover, the presence of cardiac implantable electronic device leads may introduce additional challenges in the quantitative assessment of TR severity. Large-scale prospective studies and standardization of quantitative imaging protocols are therefore needed to establish reliable and clinically applicable prognostic thresholds.

Importantly, identifying patients at increased risk of adverse outcomes does not necessarily identify those most likely to benefit from intervention. Therapeutic decision-making requires a broader assessment integrating TR severity, right-heart remodelling and function, clinical status, and procedural considerations. Accordingly, quantitative echocardiographic parameters should contribute to an integrated clinical evaluation rather than serve as isolated criteria for treatment indication or timing.

## 5. Right Atrial Function, Right Ventricular Remodelling, and RV-PA Coupling

Right atrial (RA) function has emerged as a prognostic marker in patients with TR. In a cohort of 397 patients with secondary TR, Tomaselli et al. reported that reduced RA reservoir strain (<13%) and increased indexed RA volume (>48 mL/m^2^) were independently associated with all-cause mortality or heart failure hospitalization (HR 2.11 and HR 1.49, respectively) [42]. Similarly, Galloo et al. demonstrated that RA reservoir strain provided prognostic information independent of conventional measures of RV size and function in 586 patients with severe secondary TR [43]. These findings suggest that impaired RA reservoir function may provide greater prognostic insight than atrial enlargement alone, potentially reflecting reduced atrial compliance, systemic venous congestion, and altered RA–RV interaction.

RV size should be assessed using an RV-focused apical four-chamber view to minimize foreshortening. Whenever feasible, 3D echocardiography should be used for quantification of RV volumes and ejection fraction (RVEF). 3D RVEF provides a more comprehensive assessment of global RV systolic function than conventional 2D indices, including fractional area change, tricuspid annular plane systolic excursion (TAPSE), S′ velocity, RV global longitudinal strain, and RV free-wall longitudinal strain. The prognostic value of 3D RVEF may partly reflect its ability to capture changes in global RV systolic performance, including non-longitudinal components of contraction that may become particularly relevant as RV geometry and contraction patterns evolve with progressive TR. In addition, 3D-derived RV volumes and RVEF more closely approximate CMR measurements than conventional 2D echocardiography. The proposed lower limits of normal for 3D RVEF are 46% in women and 44% in men [14,44].

Conventional indices of RV systolic function are markedly influenced by the volume and pressure overload inherent to TR. As a result, these measures may lead to an overestimation of intrinsic RV contractile performance, particularly in advanced disease stages. This load dependency represents a major limitation when assessing RV function in the setting of significant TR [5]. To mitigate this issue, increasing attention has been directed toward imaging-derived surrogates of right ventricle–pulmonary artery (RV–PA) coupling, which aim to account for afterload conditions. Physiologically, RV–PA coupling describes the relationship between RV contractility and pulmonary arterial afterload, reflecting the efficiency with which the RV converts myocardial work into forward pulmonary blood flow [14].

Among non-invasive parameters, the ratio between TAPSE and pulmonary artery systolic pressure (PASP) has been the most extensively investigated echocardiographic surrogate of RV–PA coupling and has consistently demonstrated prognostic value in patients with severe secondary TR. In the multicentre EuroTR registry, TAPSE/PASP was associated with 1-year mortality when analysed as a continuous variable (HR 0.11, 95% CI 0.04–0.29; *p* < 0.01); while a separate ROC analysis identified an optimal prognostic threshold of approximately 0.41 mm/mmHg. When patients were stratified according to RV–PA coupling tertiles (<0.32, 0.32–0.46, and >0.46 mm/mmHg), 1-year survival progressively decreased with lower TAPSE/PASP ratios (70%, 81%, and 87%, respectively) [45].

Beyond risk stratification, RV–PA coupling may also inform therapeutic decision-making. In the EuroTR registry, TEER was associated with improved survival compared with medical therapy in patients with intermediate RV–PA coupling (TAPSE/PASP 0.32–0.46 mm/mmHg; HR 0.54, 95% CI 0.31–0.94; *p* = 0.03), whereas no statistically significant benefit was observed in those with low (<0.32 mm/mmHg; HR 1.04, CI 0.68–1.61; *p* = 0.85) or high (>0.46 mm/mmHg; HR 0.69, 95% CI 0.35–1.36; *p* = 0.27) coupling. These findings suggest that RV–PA coupling may help refine patient selection, but the absence of a statistically significant survival benefit in patients with severely impaired coupling should not be interpreted as evidence of therapeutic futility [45], as symptomatic alleviation may still be achievable with interventional TR treatment.

More recently, the ratio between the absolute value of RV free-wall longitudinal strain (RVFWLS) and PASP has emerged as an alternative echocardiographic surrogate of RV–PA coupling. An |RVFWLS|/PASP ratio < 0.42%/mmHg was independently associated with an increased risk of death or heart failure hospitalization (HR 3.10, 95% CI 1.26–7.84; *p* = 0.01) [46].

Despite their proven prognostic value, interpretation of both TAPSE/PASP and |RVFWLS|/PASP depends on the accuracy of Doppler-derived PASP estimation, which may become less reliable in patients with massive, or torrential TR [46,47]. Large regurgitant orifices create low-flow conditions in which the assumptions underlying the simplified Bernoulli equation are less valid, while rapid systolic equalization of RV and RA pressures further compromises pressure estimation. In addition, RA pressure in this setting is highly dynamic and frequently underestimated when derived from inferior vena cava parameters, further compromising the accuracy of non-invasive PASP assessment [14,48]. These limitations have stimulated interest in novel indices of RV–PA coupling. Among them, the 3D echocardiographic ratio of RV forward stroke volume to RV end-systolic volume (RV forward SV/ESV) has emerged as a promising marker, integrating volumetric assessment of RV performance without relying on Doppler-derived pressure estimates. In patients with moderate or severe secondary TR, an RV forward SV/ESV ratio < 0.40 was independently associated with an increased risk of all-cause mortality or heart failure hospitalization (HR 3.36, 95% CI 1.49–7.56; *p* < 0.01). Moreover, RV forward SV/ESV showed superior prognostic discrimination compared with RVEF (area under the curve [AUC] 0.85 vs. 0.73; *p* = 0.03) and carried a higher associated risk than |RVFWLS|/PASP < 0.42%/mmHg (HR 3.10, 95% CI 1.26–7.84) and TAPSE/PASP < 0.36 mm/mmHg (HR 2.69, 95% CI 1.29–5.58) [46]. However, these findings require confirmation in larger prospective studies, including direct validation against invasive measures of RV–PA coupling. The superior prognostic performance of this index may reflect its ability to relate effective forward flow to residual RV volume, thereby capturing the interplay between ventricular remodelling and haemodynamic efficiency that may not be fully represented by conventional measures of RV systolic function.

Clinical implementation may also be constrained by the time required for 3D image analysis and the need for adequate acoustic windows to obtain reliable RV volumetric measurements.

Collectively, these findings support a shift from assessing RV systolic function in isolation toward a more integrated evaluation of RV adaptation to afterload. However, the ability of RV–PA coupling indices to predict adverse outcomes should be distinguished from their ability to identify patients most likely to benefit from TR intervention. Observational findings suggest a potential therapeutic window at intermediate stages of disease, when right-heart dysfunction is present but not yet advanced. Nevertheless, patients at either end of the RV–PA coupling spectrum should not be considered unsuitable for intervention solely on the basis of these indices. In symptomatic patients, the potential benefits of treatment should be assessed individually by the Heart Team, considering the patient’s overall clinical condition, life expectancy, procedural feasibility, and expected clinical benefit.

## 6. Echocardiography in the Transcatheter Era

### 6.1. Echocardiography-Guided Patient Selection for Transcatheter Intervention

Transcatheter tricuspid valve interventions have demonstrated significant improvements in symptoms and quality of life, although a survival benefit has not yet been established. In TRILUMINATE Pivotal (*n* = 572), TEER improved the hierarchical composite endpoint at 1 year (win ratio 1.84; *p* < 0.0001), predominantly through improvements in quality of life, without a significant reduction in mortality or heart failure hospitalizations [49]. At 2 years, recurrent heart failure hospitalizations were reduced (HR 0.72; *p* = 0.02), while mortality remained similar between groups [50]. In TRISCEND II (*n* = 400), TTVR also improved the hierarchical composite endpoint at 1 year (win ratio 2.02, 95% CI 1.56–2.62; *p* < 0.001), primarily through symptomatic and quality-of-life benefits, without a demonstrated survival advantage [51].

The emergence of transcatheter tricuspid valve interventions has expanded the role of echocardiography beyond TR diagnosis and severity grading towards the prediction of procedural success and clinical outcome. Echocardiography is central to Heart Team decision-making, integrating valve anatomy and RV remodelling to guide the timing and selection of transcatheter intervention.

**Figure 3:**
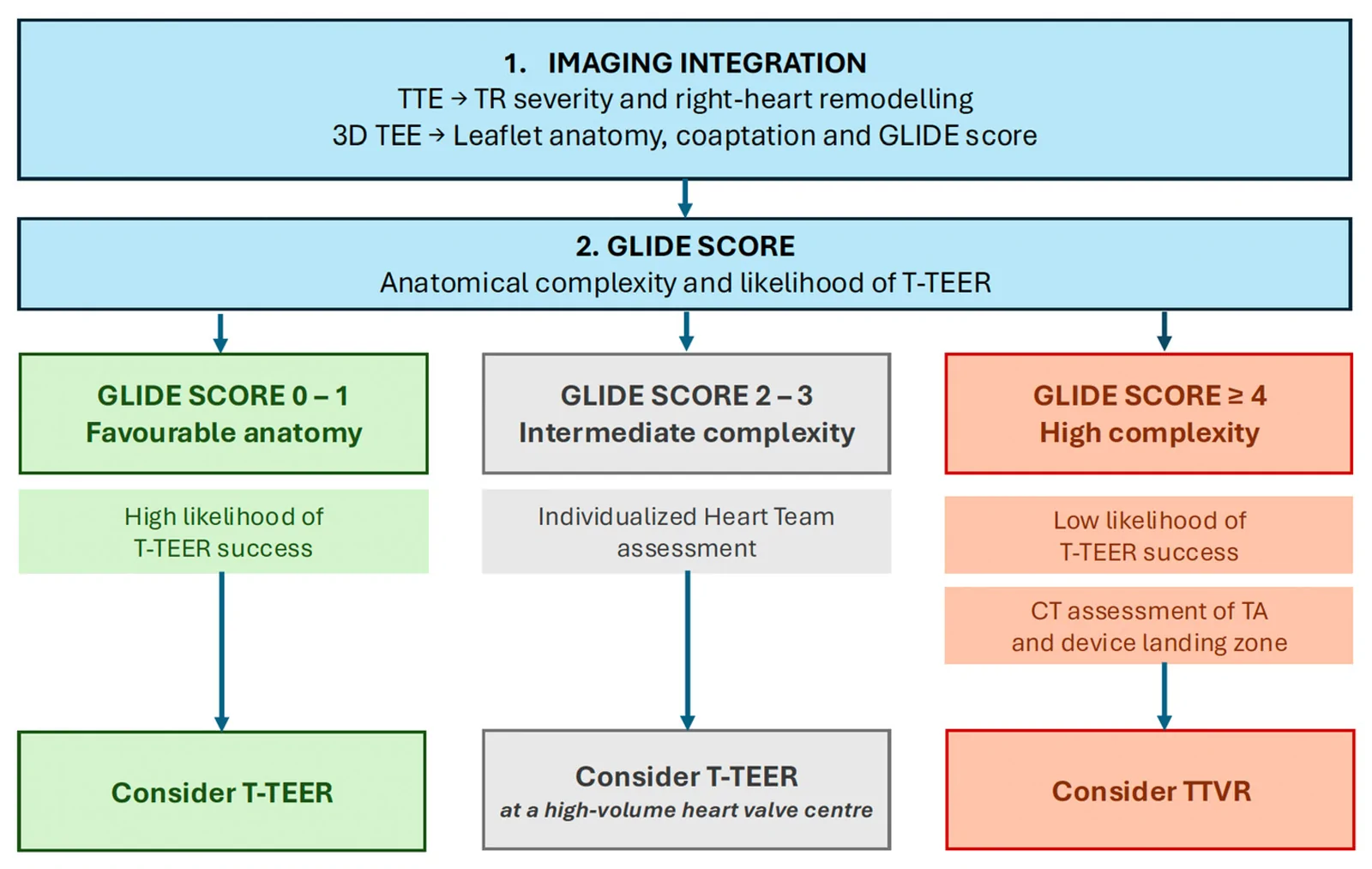
Echocardiography-guided selection of transcatheter tricuspid valve interventions. Adapted from Kempton H et al. [52].

For TEER, favourable anatomy is characterized by a small septo-lateral coaptation gap (≤7 mm), central or anteroseptal jet location, adequate leaflet length and mobility, limited tethering, and adequate echocardiographic visualization [14]. Conversely, a large coaptation gap, advanced leaflet tethering—including a tenting area > 3.15 cm^2^ or tenting height > 10 mm—more complex leaflet anatomy (>3 leaflets or scallops), unfavourable jet location, dense or complex subvalvular anatomy, and poor leaflet visualization increase the likelihood of significant residual TR after TEER. Adequate transoesophageal imaging windows are therefore themselves an important determinant of procedural feasibility. Among these features, the septo-lateral coaptation gap remains one of the most robust predictors of successful repair, with progressively larger gaps associated with a lower probability of achieving meaningful TR reduction [14,25,52]. The GLIDE score (Gap, Location, Image quality, chordal Density, and En-face TR jet morphology) combines five transesophageal echocardiographic features to predict the likelihood of successful TEER. Procedural success, defined as residual TR ≤moderate, exceeds 90% for scores of 0–1, decreases to approximately 47–61% for scores of 2–3, and falls to approximately 14–17% for scores ≥ 4 [53]. Although these findings may support consideration of alternative treatment strategies in patients with unfavourable anatomy, the association between GLIDE score and long-term clinical outcomes remains to be established (Figure 3). Regarding transcatheter tricuspid valve replacement (TTVR), imaging requirements differ from those for TEER. While TEER feasibility depends primarily on leaflet anatomy, coaptation geometry, and image quality, TTVR requires accurate assessment of the tricuspid annulus and device landing zone. Echocardiography contributes to initial anatomical evaluation, whereas CT remains essential for definitive prosthesis sizing and procedural planning [54].

Ultimately, echocardiographic findings should be integrated with the patient’s clinical status and laboratory parameters to provide a comprehensive assessment of disease severity and guide therapeutic decision-making. In this context, the TRI-SCORE (0–12 points) was originally developed to predict in-hospital mortality after isolated tricuspid valve surgery, incorporating markers of right-sided congestion, end-organ dysfunction, and RV impairment [55]. Its prognostic value has subsequently been extended to patients undergoing transcatheter interventions. In the observational TRIGISTRY registry, 2-year survival following transcatheter repair was 87%, 71%, and 56% in patients with low (≤3), intermediate (4–5), and high (≥6) TRI-SCORE values, respectively [56]. In the low TRI-SCORE category, survival rates were higher in transcatheter group than in the conservative management group (87%, and 79%, respectively, *p* = 0.001). Notably, in patients with high TRI-SCORE values (≥6), reflecting a more advanced stage of disease, successful transcatheter repair was not associated with a survival benefit compared with conservative management (adjusted HR 0.98, 95% CI 0.68–1.43; *p* = 0.90).

These findings support a comprehensive approach to patient selection for TR intervention, integrating echocardiographic assessment of procedural feasibility with clinical status and disease stage. Patients with symptomatic severe TR, favourable anatomy for transcatheter repair, and a low or intermediate TRI-SCORE may represent those most likely to achieve both effective TR reduction and meaningful clinical benefit following transcatheter intervention. Conversely, a high TRI-SCORE should not automatically be interpreted as indicating procedural futility, particularly in patients for whom symptomatic improvement may remain a relevant therapeutic goal despite the absence of a demonstrated survival benefit. Ultimately, a multidisciplinary Heart Team approach integrating echocardiographic findings with clinical assessment may help identify the most appropriate treatment strategy.

Treatment pathways are illustrative and require individualized Heart Team assessment.

### 6.2. Post-Intervention Echocardiographic Assessment

Accurate assessment of residual TR is particularly relevant from a prognostic perspective. In the EuroTR registry, residual severe or greater TR (≥3+) after transcatheter edge-to-edge repair was independently associated with increased mortality (HR 2.06, 95% CI 1.30–3.26; *p* = 0.002) [57]. Similarly, an analysis from the TRIGISTRY registry demonstrated a progressive decline in 2-year survival according to residual TR severity, from 85% in patients with mild or less severe TR to 70% and 44% in those with moderate and severe residual TR, respectively [56]. These findings suggest that procedural success should not be defined solely by a reduction in TR grade, but rather by the absolute severity of residual regurgitation, ideally achieving mild or less TR.

However, grading residual TR after intervention remains challenging and requires an integrated multiparametric approach [9]. Leaflet approximation alters regurgitant orifice geometry, frequently generates multiple residual jets, and distorts proximal flow convergence. Consequently, conventional semi-quantitative and quantitative parameters, including 2D VCW and PISA-derived effective regurgitant orifice area and regurgitant volume, become less reliable and may underestimate residual TR severity. 3D-VCA may overcome some of these geometric limitations by directly quantifying the residual regurgitant orifice and has emerged as a useful parameter for assessing changes in TR severity following intervention. Device position and function should also be systematically assessed, together with the transvalvular gradient, although increased post-TEER gradients have not been associated with adverse clinical outcomes [9]. Following TTVR, echocardiographic assessment should include prosthetic valve function, residual TR, particularly paravalvular regurgitation, and transvalvular gradients. Changes in mitral regurgitation should also be evaluated, as TR elimination and the resulting haemodynamic changes may be associated with worsening mitral regurgitation in some patients. Beyond assessment of the immediate procedural result, serial echocardiography provides additional prognostic information by tracking changes in RV size and function, RA and TA dimensions, and the extent of right-heart reverse remodelling over time [14].

## 7. Future Perspectives

Despite substantial advances in TR imaging, several important gaps remain. Current aetiological classification into atrial and ventricular functional TR has improved phenotyping and prognostic stratification, but substantial heterogeneity likely exists within these categories. A-FTR may encompass different patterns of leaflet malcoaptation, ranging from relatively symmetric leaflet restriction with a central regurgitant jet to asymmetric restriction predominantly involving the septal leaflet, analogous to phenotypes recently described in atrial functional mitral regurgitation. Further phenotypic characterization is therefore needed to determine whether these subgroups have distinct natural histories, prognoses, and responses to intervention. TR quantification also remains challenging because of its complex geometry, dynamic nature, and load dependency, with no single echocardiographic parameter providing an adequate reference standard. Automated or artificial intelligence-based assessment of parameters such as 3D-VCA or PISA-derived measurements may improve reproducibility and facilitate quantitative assessment. Similar challenges apply to 3D evaluation of RV function and TA geometry, for which dependence on image quality and interobserver variability continues to limit widespread routine implementation. Further refinement of right-heart assessment is also needed. Beyond TAPSE/PASP, novel non-invasive indices of RV–pulmonary artery coupling should be developed and validated against invasive haemodynamic measurements, particularly in advanced TR, in which Doppler-derived estimates of pulmonary pressures may be unreliable. Most importantly, imaging should evolve from predicting anatomical feasibility towards identifying the optimal timing and strategy for intervention according to the stage of right-heart remodelling. Beyond the GLIDE score, additional imaging markers integrating RV function, and pulmonary haemodynamics may improve selection between TEER and TTVR and identify patients most likely to derive meaningful clinical benefit. Finally, standardized imaging endpoints after intervention remain needed, including the optimal definition of successful residual TR reduction and the magnitude of right-heart reverse remodelling, which should be considered a meaningful treatment response.

## 8. Conclusions

Echocardiographic assessment of TR is evolving towards a more comprehensive evaluation integrating disease mechanism, detailed assessment of tricuspid annular geometry, advanced quantitative methods for TR severity assessment, right-heart remodelling, and right ventricular–pulmonary arterial coupling. Traditionally, echocardiographic research has focused primarily on identifying and validating prognostic markers, as reflected in the expanded five-grade classification of TR severity. However, beyond risk stratification, greater emphasis is needed for identifying patients most likely to benefit from intervention and determining its optimal timing. This requires integrating echocardiographic predictors of procedural success, such as the GLIDE score, with clinical indicators of disease progression and end-organ dysfunction, as captured by the TRI-SCORE. Ultimately, these parameters should inform, rather than replace, individualized Heart Team decision-making, taking into account the patient’s overall clinical condition, life expectancy, procedural feasibility, and expected clinical benefit.

## Data Availability

No new data were created or analyzed in this study. Data sharing is not applicable to this article.

## Abbreviations

The following abbreviations are used in this manuscript:

2D	two-dimensional
3D	three-dimensional
4D-CT	four-dimensional computed tomography
A-FTR	atrial functional tricuspid regurgitation
AUC	area under the curve
CIED	cardiac implantable electronic device
CMR	cardiac magnetic resonance
CT	computed tomography
EROA	effective regurgitant orifice area
ESV	endsystolic volume
GLIDE	gap, location, image quality, chordal density, and en-face TR jet morphology
HF	heart failure
HFpEF	heart failure with preserved ejection fraction
HFrEF	heart failure with reduced ejection fraction
LVEF	left ventricular ejection fraction
PASP	pulmonary artery systolic pressure
PISA	proximal isovelocity surface area
RA	right atrium
RVol	regurgitant volume
RV	right ventricle
RVEF	right ventricular ejection fraction
RVFWLS	right ventricular free-wall longitudinal strain
RV–PA	right ventricular–pulmonary artery
SV	stroke volume
TA	tricuspid annulus
TAPSE	tricuspid annular plane systolic excursion
TEE	transesophageal echocardiography
TEER	transcatheter edge-to-edge repair
TR	tricuspid regurgitation
TTE	transthoracic echocardiography
TTVR	transcatheter tricuspid valve replacement
VCA	vena contracta area
VCW	vena contracta width
V-FTR	ventricular functional tricuspid regurgitation
